# Stress distribution pattern of screw-retained restorations with segmented vs. non-segmented abutments: A finite element analysis

**DOI:** 10.15171/joddd.2017.027

**Published:** 2017-09-20

**Authors:** Shima Aalaei, Zahra Rajabi Naraki, Fatemeh Nematollahi, Elaheh Beyabanaki, Afsaneh Shahrokhi Rad

**Affiliations:** ^1^Dental Caries Prevention Research Center, Qazvin University of Medical Sciences, Qazvin, Iran; ^2^Private Practice, Qazvin, Iran; ^3^Department of Prosthodontics, Islamic Azad University, Dental Branch, Tehran, Iran; ^4^Department of Prosthodontics, School of Dentistry, Shahid Beheshti University of Medical Sciences, Tehran, Iran; ^5^Department of Restorative Dentistry and Biomaterials Sciences, Harvard School of Dental Medicine, USA

**Keywords:** Antibacterial, biofilm, *Enterococcus faecalis*, sodium hypochlorite

## Abstract

***Background.*** Screw-retained restorations are favored in some clinical situations such as limited inter-occlusal spaces. This study was designed to compare stresses developed in the peri-implant bone in two different types of screw-retained restorations (segmented vs. non-segmented abutment) using a finite element model.

***
Methods.*** An implant, 4.1 mm in diameter and 10 mm in length, was placed in the first molar site of a mandibular model with 1 mm of cortical bone on the buccal and lingual sides. Segmented and non-segmented screw abutments with their crowns were placed on the simulated implant in each model. After loading (100 N, axial and 45° non-axial), von Mises stress was recorded using ANSYS software, version 12.0.1.

***
Results.*** The maximum stresses in the non-segmented abutment screw were less than those of segmented abutment (87 vs. 100, and 375 vs. 430 MPa under axial and non-axial loading, respectively). The maximum stresses in the peri-implant bone for the model with segmented abutment were less than those of non-segmented ones (21 vs. 24 MPa, and 31 vs. 126 MPa under vertical and angular loading, respectively). In addition, the micro-strain of peri-implant bone for the segmented abutment restoration was less than that of non-segmented abutment.

***
Conclusion.*** Under axial and non-axial loadings, non-segmented abutment showed less stress concentration in the screw, while there was less stress and strain in the peri-implant bone in the segmented abutment.

## Introduction

Implant-supported prostheses have been widely used in recent decades because of their promising esthetic, functional and biological outcomes.^1^ However, the long-term success of dental implants is affected by several factors, including implant biomechanics, distribution of load at the bone‒implant interface, and stress transfer to the bone.^2-5^

Implant-supported prostheses are categorized into two major types; screw-retained and cement-retained restorations. Although each type of restoration has some advantages,^6,7^ their selection is mostly based on the clinician’s preference.^8^ According to Heckmann et al^9^ there is no difference between the precision of fit of these two types of restorations. Moreover, the stress developed in the peri-implant bone supporting screw-retained and cement-retained restorations was reported to be similar.^9^

Screw-retained restorations have several advantages over cement-retained ones, including retrievability, higher stability and security of implant‒abutment connection.^10^ Therefore, it is highly recommended that such restorations be used in clinical situations such as subgingival margins of restorations deeper than 3 mm,^11^ limited inter-occlusal space (less than 4 mm)^12^ and where biological or technical complications are anticipated.^13^ Furthermore, due to the retrievability characteristics, screw-retained restorations are also recommended for cantilever restorations.^14^

Screw-retained restorations can be fabricated with two types of abutments: segmented and non-segmented abutments.^15^ In a non-segmented abutment, the restoration is directly fabricated and connected to the implant, which can create a more desirable emergence profile and esthetics when there is limited inter-occlusal space available.^16^ Another advantage of non-segmented abutments is the reduced number of abutment components which can reduce the complications and the cost of restoration.^17^

Since stress distribution in the peri-implant bone is a critical factor for the success of implant-supported prostheses,^17,18^ several studies have been conducted to investigate stress distribution in the implant components and the surrounding bone by using finite element analysis (FEA).^19-33^ Considering the possible role of abutment and prosthesis design in stress distribution in the restoration, and the stresses transferred to the bone, the comparative effect of segmented and non-segmented abutments on stress transfer is unknown. Theoretically, given a decrease in the number of screws and the micro-motion of the components in the non-segmented abutments, the amount of stresses transferred to the bone would increase. There is no study investigating the stress distribution pattern in different types of abutments and peri-implant bone. Therefore, the aim of this study was to evaluate and compare the stress distribution pattern in the crestal bone around the implants supporting the restorations of segmented and non-segmented screw abutments, and also stress distribution pattern in the abutment screws.

## Methods

### FEA model design

Computed tomographic (CT) images of an edentulous mandible at the first molar region at 0.5-mm intervals of an adult male were used to develop a 3D model by means of modeling software (Solid Works Corp., Concord, MA, USA). Two separate 3D models of a dental implant (Straumann, regular neck tissue level, Institut Straumann AG, Basel, Switzerland), 10 mm in length and 4.1 mm in width at the body and 4.8 mm at the platform, were simulated in Solid Works 2010 from measurements acquired by a profile projector (Microtechnical LTF, Italy) with 0.01-mm accuracy. The implants were placed in bone with 1-mm-thick cortical bone on the buccal and lingual sides over the cancellous core.^33^

A segmented abutment (RNSynocta 1.5, 1.5-mm height, Straumann, Switzerland) with a regular neck Synocta Gold coping for Synocta 1.5, 4.25-mm/12-mm height, and a non-segmented abutment (RNSynocta Gold Abutment, 4.3-mm height, Straumann, Switzerland) were also modeled to support the crowns. A symmetrical porcelain-fused-to-metal crown (12 mm mesiodistally and 9 mm buccolingually) was simulated using a high-noble alloy for the framework and 1-mm uniform thickness of porcelain on the metal framework. The same size of crown was modeled for the non-segmented abutment, with a difference that the framework was modeled as a part of the abutment, so that the crown and abutment were modeled in one piece. The crowns were designed in a way that the center of the crowns coincided to the long axis of the implants. The models were then exported to the ANSYS Workbench version 11.0 (ANSYS Inc., Canonsburg, PA, USA) for analysis. The contact between the screw and the implant and abutment was considered frictional, with bonded surfaces at all other surfaces.

**Figure 1:**
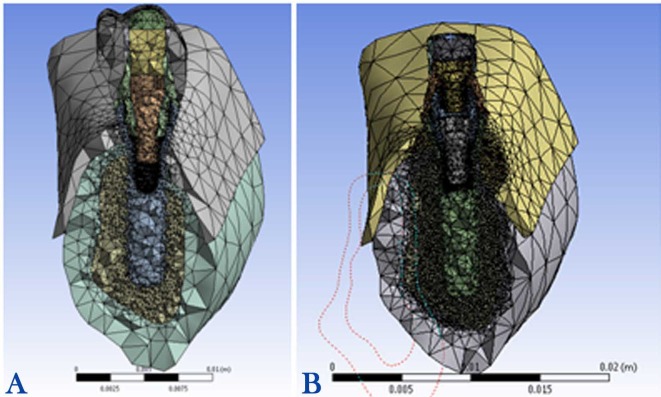
Completed meshed models of non-segmented abutment (A) and segmented abutment (B).

The completed models were meshed by parabolic tetrahedral elements (Figure 1A B and B). The model with the segmented abutment incorporated 233,057 elements and 373,095 nodes, and the model with non-segmented abutment consisted of 201,172 elements and 325,080 nodes‏. All the nodes at the base of 3D models were restrained to determine the boundary conditions. The implants, abutments, abutment screws, crowns, and cortical and cancellous bones were considered to be homogeneous, isotropic and linearly elastic.^33^ The elastic properties of materials used in the FE model are listed in Table 1. Gold alloy (Ceramco, Dentsply Inc., York, PA, U.S.A.) and Vita porcelain (Vita Zahnfabrik, Säckingen, Germany) were used.

**Table 1:** Mechanical characteristics of studied materials

**Material**	**Modulus of elasticity (Mpa)**	**Poisson’s Ratio**
Cancellous bone	1370	0.30
Cortical bone	13700	0.30
Porcelain	69000	0.28
High-noble alloy	100000	0.30
Titanium	103400	0.33

Two loading sets of 100-N were simulated and applied at the central fossa of the crowns in two different directions vertically^34^ and obliquely (45°) relative to the long axis of the implants‏. Osseointegration was considered to be complete. The implant‒bone contact was simulated to present complete osseointegration.

### FEA data collection

ANSYS software (ANSYS WB 2.0 Framework, version 12.0.1, 2013 SAS IP) was used for quantitative and qualitative stress analyses, considering material properties, meshing and loading. The maximum von Mises stresses (maximum equivalent stress) at the implant surface and abutment screws and also strain values were reported as the qualitative analysis. The stress distribution pattern was also evaluated by color-coded diagrams as the quantitative analysis, in which areas with the highest and the lowest stresses were depicted as red and blue, respectively.

## Results

The maximum stress and Strain value in the peri-implant bone, and in segmented and non-segmented abutment screws were evaluated and compared (Table 2). In both models and under each loading condition, maximum stress concentration was detected around the neck of the implants. Moreover, as the distance from the implant increased, a decrease in peri-implant bone stress was observed. Furthermore, von Mises stress values were comparatively higher under angular loading condition.

**Table 2:** Maximum von Mises stress and strain values in the peri-implant bone and abutment screw in the finite element model

**Model**	**Maximum von Mises stress in bone**	**Maximum von Mises stress in abutment screw**	**Maximum von Mises strain in bone**
**Segmented abutment,** **axial loading**	21	100	4400
**Segmented abutment,** **non-axial loading**	31	430	9400
**Non-segmented abutment,** **axial loading**	24	87	2200
**Non-segmented abutment,** **non-axial loading**	126	375	2400

Further stress analysis showed that the micro-strain values developed in the peri-implant bone with the non-segmented abutment were higher (4400 and 9400 units under vertical and angulated loadings, respectively) than those of the segmented abutment (2200 and 2400 units under vertical and angulated loadings, respectively).

### Stress analysis for the segmented abutment

**Figure 2:**
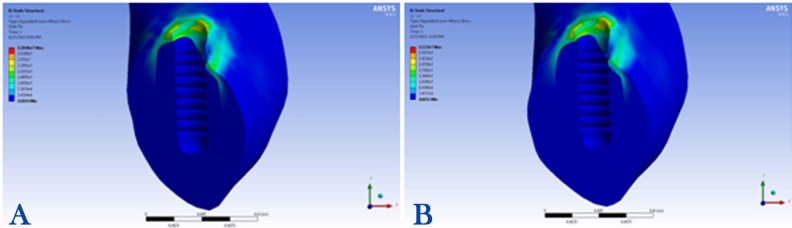
Stress distributions in the peri-implant bone of segmented abutment under vertical (A) and angular loadings (B).

**Figure 3:**
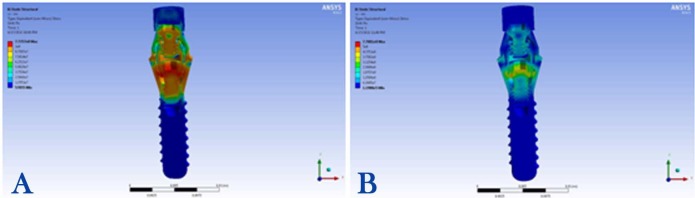
Stress distributions in the segmented abutment under vertical (A) and angular loadings (B).

1) Maximum stress concentration in the peri-implant bone was 21 MPa in the mesiolingual area under the 100-N vertical loading condition (Figure 2A).

2) Maximum stress concentration in the peri-implant bone was 31 MPa in the mesiolingual area under the 100-N angular loading condition (Figure 2B).

3) Maximum stress concentration in the abutment screw was 100 MPa detected in the neck, the first and second threads, under the 100-N vertical loading condition (Figure 3A).

4) Maximum stress concentration in the abutment screw was 430 MPa recorded in the neck, the first and second threads, under the 100-N angular loading condition (Figure 3B).

#### Stress analysis for the non-segmented abutment

**Figure 4:**
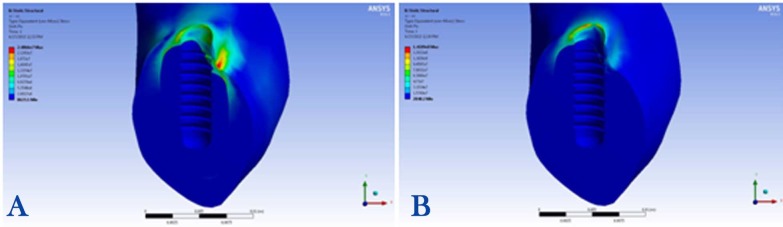
Stress distributions in the peri-implant bone of non-segmented abutment under vertical (A) and angular loadings (B).

**Figure 5:**
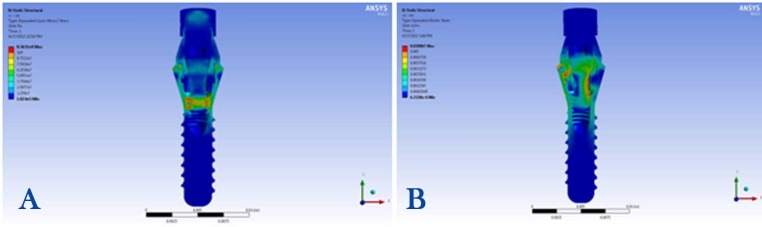
Stress distributions in the non-segmented abutment under vertical (A) and angular loadings (B).

1) Maximum stress concentration in the peri-implant bone was 24 MPa, detected in the buccal area, under the 100-N vertical loading condition (Figure 4A).

2) Maximum stress concentration in the peri-implant bone was 126 MPa, observed in the distolingual area, under the 100-N angular loading condition (Figure 4B).

3) Maximum stress concentration in the abutment screw was 87 MPa, observed in the first and second threads, under the 100-N vertical loading condition (Figure 5A).

4) Maximum stress concentration in the abutment screw was 375 MPa in the neck, observed in the first and second threads, under the 100-N angular loading condition (Figure 5B).

## Discussion

The present study was designed in an effort to compare the effect of using segmented versus non-segmented abutments on stress distribution in the peri-implant bone, and the abutment screws in screw-retained restorations using a 3-dimensional finite element analysis. The von Mises stress was used for evaluating bone stress, and determining if any bone damages would occur under a complex loading condition. The simulated bite force used in this study was 100 N,^34^ which was applied to the center of the occlusal surfaces of the crowns.

The study demonstrated that the stress concentration and microstrain in the peri-implant bone in the model with non-segmented abutment was greater than that of the segmented abutment. The results of a photoelastic study by Ochiaiet al^17^ showed that non-segmented abutments which are subjected to vertical loading create more non-lateral stress concentration in the bone as compared to the segmented abutments. According to Rangert et al,^4^ the flexibility of the implant components can give some freedom of movement, and therefore reduce stress. This finding is consistent with the results of our study, which showed reduced microstrain in peri-implant bone with the segmented abutment. This can be explained by the greater micromotion produced in the segmented abutments with two screws in comparison to segmented abutments with only one screw. Furthermore, given the greater diameter of the non-segmented abutment screw than either occlusal screw or abutment screw of the segmented abutment, it could be concluded that the effect of the number of the screws on the reduction of stresses transferred to the implant‒bone interface might be more important than the screw diameter. According to Frost’s classification of microstrain at bone‒implant interface under different loading conditions, while microstrain values of 50‒2500 are within ideal loading zone, values >4000 in the peri-implant bone are considered as pathologic overload zone.^35^ The results of the present study demonstrated that microstrain values for models with non-segmented abutment were in the pathologic overload zone, whereas these values for the segmented abutment were within ideal loading zone. From a biological aspect, it can be concluded that using segmented abutments for screw-retained restorations is more suitable in reducing bone stress and strain.

The stress produced in the segmented abutment screw under vertical loading (100 MPa) was greater than the stress value in the non-segmented abutment screw (87 MPa). This finding also confirms the previous finding that most of the stress would be concentrated in the abutment and prosthesis screws in the segmented abutment before it reaches the bone‒implant interface. As there is a high stress concentration in the segmented abutment screw compared to the non-segmented abutment screw, it seems necessary to control the over-loading conditions to avoid clinical complications such as screw loosening and/or fracture. Since the abutment screw is the weakest component of the assembly, loosening of this screw can be a good indicator of the overloading condition and to identify an overloading problem before progress to a more serious situation such as fracture of the implant (especially with internal connections) and resorption of bone. Furthermore, stress concentration at bone‒implant interface was less than that in the abutment screw.

The maximum von Mises stress was found in the collar region of the implant regardless of the type of model. This finding is consistent with the majority of other studies.^19,21,22^ If the stress produced in the crestal bone exceeds the elastic limit of bone, it results in microfarcture of the bone and subsequently in bone resorption.^5^ This emphasizes the importance of the presence of bone with good quality in the implant neck region.^30^

The results of this study also demonstrated higher stress concentration under angular loading as compared to vertical loading. Pellizzer et al^18^ and Qian et al^20^ also showed that stress concentration was greater under angular than vertical loadings. Therefore, it is recommended to reduce angular loadings as much as possible through selecting straight abutments and fabricating crowns with shallower cuspal inclination to reduce strain production in the peri-implant bone and implant‒abutment components.

According to the results of this study, segmented abutments are a better clinical choice than non-segmented ones considering less stress concentration and microstrain developed in bone. Furthermore, non-segmented abutments with chrome‒cobalt connection have some other disadvantages such as non-ideal sealing properties, probability of galvanic corrosion and consequently bone loss.^15^

One of the limitations of FEA studies is considering all materials as homogeneous and isotropic with linear elasticity; this may limit extending the results to clinical situations. In addition, all FEA studies assume 100% osseointegration at bone‒implant interface, which is not the case in biologic or clinical situations. Therefore, clinical studies are warranted to explore the effect of the selected abutments on the integrity of the bone around the implants for single- and multiple-unit prostheses.

## Conclusion

Within the limitations of the present study, it can be concluded that segmented screw abutments offer biomechanical advantages in terms of reducing stress concentration and microstrain in bone. The stress concentration in the abutment screw was higher in segmented abutment than that in the non-segmented abutment.

## Acknowledgments

The authors would like to thank the Department of Prosthodontics of Qazvin University of Medical Sciences for supporting this study.

## Authors’ contributions

SA and FN designed the study. ZRN collected the data. ASR and ZRN analyzed the data. EB and SA prepared the manuscript. All authors have read and approved the final manuscript.

## Funding

The study was funded by Qazvin University of Medical Sciences.

## Competing interests

The authors declare no competing interests with regards to the authorship and/or publication of this article.

## Ethics approval

This study approved by ethics committee of Qazvin University of Medical Sciences (ethics reference number IR.QUMS.REC.1394.474).
